# Anti-Cancer Effects of Quercetin: What Role Does the Gut Microbiota Play?

**DOI:** 10.3390/molecules31091456

**Published:** 2026-04-28

**Authors:** Guang Yi, Yang Liu, Guangye Li, Ke Chang

**Affiliations:** School of Clinical Medicine, Chengdu University of Traditional Chinese Medicine, Chengdu 611137, China; cdzyyyg@163.com (G.Y.);

**Keywords:** quercetin, gut microbiota, cancer, phenolic acid, interaction

## Abstract

Cancer threatens the health and lives of people around the world and causes a heavy economic burden on families and society. Dysbiosis of the gut microbiota has been observed in cancer patients and is an important factor in cancer progression. Quercetin, a widely distributed dietary flavonol, exhibits pleiotropic anti-cancer activities in preclinical models. Importantly, recent studies reveal a bidirectional crosstalk between quercetin and the gut microbiota that may critically shape its biological effects. Specifically, gut microbiota enzymes mediate quercetin biotransformation and produce tumor-suppressive quercetin metabolites. On the other hand, quercetin remodels specific gut microbial species and their metabolites to promote anti-tumor activity. This review provides a timely and systematic synthesis of the latest findings regarding quercetin, gut microbiota, and cancer. Furthermore, we discuss strategies to enhance this interaction for improved cancer therapy. By highlighting the pivotal role of the gut microbiota, this review offers novel insights and a refined theoretical framework to guide future research and potential clinical translation of quercetin in cancer prevention and treatment.

## 1. Introduction

Cancer is caused by multiple etiological factors and is primarily characterized by uncontrolled cellular proliferation and the behavior of invasion and metastasis [1]. In recent decades, cancer research has received heightened attention due to being the leading cause of human death and the economic burden it imposes on society [2]. However, traditional cancer treatments, including surgery, chemotherapy, radiotherapy, and immunotherapy, are constrained by challenges such as adverse effects and drug resistance [3,4]. Therefore, the development of more efficient or combined therapies to overcome these challenges is of great significance for cancer patients.

The human body harbors trillions of microorganisms, including bacteria, viruses, fungi, and protozoa, with their population approximately equating to that of the host cells [5,6]. Among them, the gut microbiota emerges as a hot topic of scientific research due to its critical role in maintaining normal physiological activities of the body. For this reason, the gut microbiota is also known as “the hidden organ” [7,8]. At the same time, dysbiosis of the gut microbiota has been reported to be closely associated with the occurrence and development of numerous diseases, especially cancer. With the advancement of detection technology, the underlying mechanisms of gut microbiota influencing cancer have been gradually revealed [9,10]. Moreover, experiments have also demonstrated that restoring the balance of the gut microbiota can effectively promote the outcomes of cancer treatment through drug biotransformation and synergistic anti-cancer effects [11,12].

Quercetin (3,3′,4′,5,7-pentahydroxyflavone) belongs to flavonoids classified within the polyphenol family and is present in a diverse array of dietary plants [13]. Both in vitro studies and animal models have revealed the direct antitumor effects of quercetin, with the specific mechanisms being extensively elucidated [14]. Notably, a portion of ingested quercetin reaches the colon, where it interacts with the gut microbiota [15,16,17,18]. The gut microbiota metabolizes quercetin into phenolic acids with highly anti-cancer activity, and, in turn, quercetin can drive the transformation of gut microbiota toward an anti-cancer phenotype. Currently, a growing body of research is gradually revealing the related phenolic acid metabolites and the bacterial species influenced by quercetin. Nevertheless, a corresponding review that comprehensively delineates this overall landscape remains notably absent. Highlighting and improving the role of the gut microbiota in the anti-cancer effect of quercetin has opened new avenues for clinical application.

This review will systematically summarize the influence of the interaction between quercetin and gut microbiota on tumor cells. Firstly, we outline the metabolic processes and anti-cancer effects of quercetin. Next, we describe the characteristics of gut microbiota imbalances in cancer patients and common modifications of gut microbiota. We then summarized the interaction between quercetin and gut microbiota from two aspects: (a) the metabolism of quercetin by gut microbiota and the anti-cancer mechanism of phenolic acids, and (b) how changes in gut microbiota structure and metabolites after quercetin treatment support cancer treatment and prevention. Based on the above process, we also propose the strategies of enhancing the interaction between quercetin and gut microbiota. These findings complement the perspective on the gut microbiota in quercetin’s anti-cancer therapy, providing theoretical support and guidance for further research.

## 2. Literature Search Strategy and Study Selection

This narrative review was conceptualized with reference to the Scale for the Assessment of Narrative Review Articles (SANRA) guidelines, though it was not formulated in strict adherence to these criteria. To compile this review, we systematically searched the most widely used and comprehensive biomedical databases, including PubMed, Embase, Web of Science, and Cochrane Library. Time Window (start and end dates): The literature search encompassed all relevant studies published from the inception of each database up to December 2025. Inclusion/Exclusion Criteria: Keywords such as “quercetin”, “quercetin metabolites/phenolic acids”, “flavonoids”, “flavonols”, “polyphenols”, “gut microbiota”, “gut microbiome”, “cancer”, “neoplasms”, “tumors”, “colorectal neoplasms”, “gastrointestinal microbiome”, “breast neoplasms”, “quercetin and cancer”, and “quercetin and gut microbiota/microbiome” were used for database searching. The search strategy employed controlled vocabulary terms and free-text keywords combined with Boolean operators. The core search string was structured as: (quercetin OR “quercetin glycoside”) AND (“gut microbiota” OR microbiome OR “intestinal flora”) AND (cancer OR neoplasm OR tumor). No publication date or language restrictions were initially applied. Studies were excluded if they were preprints or case reports (with <5 cases), lacked extractable data, used unvalidated methods, were irrelevant to the topic, or were duplicate publications. Study Selection Process: Database-derived articles underwent a three-step selection process: first, duplicate removal via reference management tool Mendeley; second, title and abstract screening to filter out irrelevant studies; and third, full-text retrieval and eligibility assessment in accordance with the preset inclusion and exclusion criteria. Quality/Risk-of-Bias Assessment: Formal quality or risk-of-bias assessment was not performed, as this review is intended to offer a broad perspective on the existing evidence and summarize key advances in the interplay between quercetin, gut microbiota, and cancer, rather than a rigorous systematic review or meta-analysis. However, the absence of such an assessment means we cannot systematically rule out the potential influence of studies with lower methodological quality (e.g., small sample size, lack of control groups, incomplete outcome reporting) on the overall findings. This could potentially lead to an overestimation of the strength or consistency of the effects of the quercetin-gut microbiota interaction, thereby somewhat attenuating the certainty of the conclusions drawn herein. To safeguard the credibility of the included studies, we focused on selecting high-quality publications with transparent methodologies and extractable data. Future research should address this issue through systematic reviews and meta-analyses that incorporate rigorous risk-of-bias assessments for a more quantitative synthesis.

## 3. Quercetin, Metabolic Processes, and Anti-Cancer Effects

**Figure 1:**
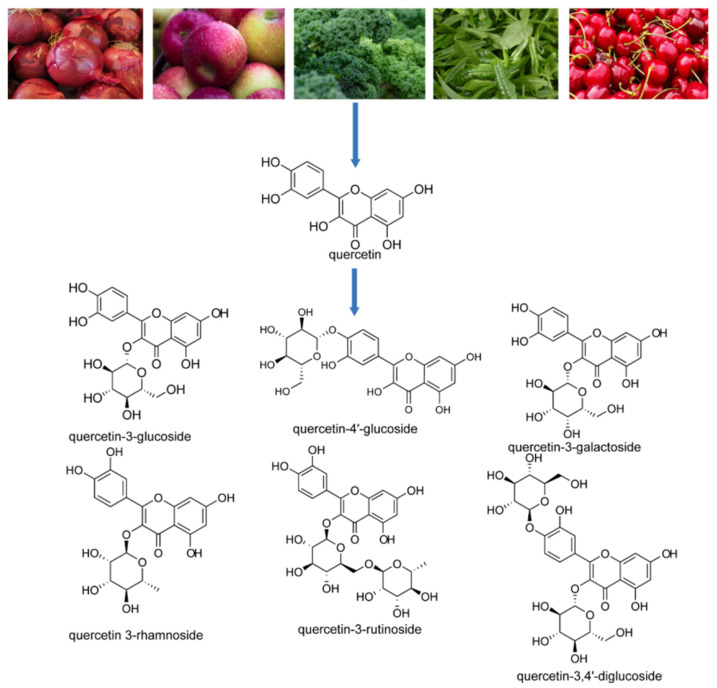
Foods rich in quercetin in nature and their corresponding structures.

The anti-cancer effects of quercetin are largely constrained by its metabolic processes and pharmacokinetic properties. Initially, quercitrin needs to be liberated from the food matrix and persist in the gastrointestinal lumen to be available for absorption, which is termed “bioaccessibility”. However, due to its low solubility and poor stability, quercitrin exhibits low bioaccessibility (bioaccessibility index < 1). To address this limitation, various delivery systems and in vitro fermentation technologies have been developed, enabling targeted release and significantly enhanced BI [19,20,21]. The released quercetin subsequently undergoes absorption, metabolism, and distribution. Ultimately, only a portion of the quercetin enters systemic circulation and reaches the target tissues, a process quantified as bioavailability. The bioavailability of quercetin is closely related to the metabolic process, which is the basis for the selection of quercetin intervention level in vitro experiments and the basis for improving the biological effect of quercetin in vivo. Multiple determination methods reveal that natural quercetin usually forms conjugates with sugar moieties, collectively known as quercitrin (Figure 1) [22,23,24,25]. In contrast to quercetin, which can be partially absorbed and metabolized by the stomach, quercetin glycosides are broken down by glycosidases produced by intestinal epithelial cells and gut microbiota [26]. Subsequently, released quercetin aglycones enter the intestinal epithelium via passive diffusion or organic anion transporting polypeptide [15,27,28,29]. Researchers also discovered that specific quercetin glycosides, such as Q3G, are previously transported into intestinal epithelial cells by glucose transporters and then hydrolyzed via cytosolic β-glucosidase (CBG) [27,30]. After absorption, quercetin undergoes phase II metabolism that has been investigated using mouse models and human intestinal and liver in vitro models. The results showed that the phase II metabolism of quercetin mainly occurred in the small intestine and liver, including glucuronidation, methylation, and sulfation [31,32]. Among them, the small intestine mostly contributes to the sulfation of quercetin, exhibiting significant variability among individuals. The produced quercetin metabolites may be either released into the bloodstream or excreted into the intestinal lumen with bile. Therefore, the level and activity of these enzymes that facilitate phase II metabolism and the rate of biliary excretion significantly affect the plasma concentration and bioavailability of quercetin [33]. Furthermore, the involvement of gut microbiota in the metabolic processing of quercetin glycosides is becoming increasingly elucidated. In the colon, the remaining quercetin glycosides, quercetin aglycones, and quercetin conjugates secreted with bile go through a multiple-step biotransformation to finally produce phenolic acids by the gut microbiota [15,34]. The relevant enzymes and microbial species are discussed in detail in the following sections. Eventually, quercetin-derived metabolites are excreted in feces or urine [35]. Overall, the comprehensive elucidation of the metabolic process of quercetin suggests potential strategies for enhancing its bioavailability [16]. Based on the role of gut microbiota, recent studies have concentrated on modulating the gut microbiota as a means to decrease the required dosage of quercetin for treatment purposes and address the variability observed among individuals. This approach is favored due to its potential for more straightforward detection and regulation.

The anti-cancer effects of quercetin have been confirmed in multiple studies. Decades ago, it was widely held that the antioxidant properties of quercetin could effectively prevent the occurrence of cancer [36]. Experiments also further revealed that quercetin induces direct apoptosis in cancer cells and disrupts their adaptive resistance mechanisms by moderately attenuating oxidative stress, thereby suppressing subsequent inflammatory pathways and other cancer-promoting signaling pathways [37,38]. Excitingly, more direct anticancer mechanisms of quercetin have been revealed, including inhibition of proliferation, induction of apoptosis, reduction of metastasis, and stimulation of anti-cancer immune responses [39,40]. Over the past few years, with the advancement of drug delivery technologies, a growing number of in vivo experiments have been conducted, which have further revealed the therapeutic application potential of quercetin (Table 1). For example, Lin et al. designed a self-assembled nanoparticle in which quercetin is encapsulated by fucoidan, with tea saponin serving as the linker between these two components. Compared with free quercetin, the nanoparticles exhibited higher inhibitory activity against tumor proliferation and migration in human non-small cell lung cancer A549 tumor-bearing mice [41]. Notably, owing to its diverse anti-cancer effects, quercetin can augment the efficacy of conventional cancer therapies and partially overcome resistance [42,43,44]. However, the anti-cancer function of quercetin lacks strong clinical evidence. A study reported that five patients with familial adenomatous polyposis who underwent colectomy experienced a 60.4% reduction in polyp count (*p* < 0.05) and a 50.9% decrease in polyp size (*p* < 0.05) after six months of treatment with curcumin and quercetin [45]. For cancer prevention, two observational studies found that quercetin intake may reduce the incidence of ovarian cancer and gastric cancer [46,47]. It is important to emphasize that these findings are derived from studies with small sample sizes or observational designs, and therefore constitute very limited evidence; they are not sufficient to form the basis for clinical recommendations. Confirmation through large-scale, randomized, double-blind, controlled trials is required. In the future, the clinical application of quercetin will need to overcome the problem of low bioavailability. Undeniably, quercetin as an adjuvant for cancer therapy is a promising option. Moreover, no clinical studies have involved the measurement of gut microbiota and quercetin-derived metabolite levels, which may be important contributors to the differences in therapeutic efficacy. In the future, delivery systems and gut microbiota are expected to facilitate the implementation of more high-quality clinical trials. Besides, clinical trials of quercetin still require safety assessment. Preclinical studies generally indicate a favorable safety profile for quercetin, with subchronic and chronic toxicity studies reporting no significant adverse effects on organ function or metabolism, and in vivo genotoxicity assays typically yielding negative results [48,49,50]. However, a phenomenon termed the “quercetin paradox” complicates this assessment: the oxidation of quercetin generates reactive ortho-quinone and quinone-methide intermediates, which exhibit high reactivity toward thiol groups, potentially leading to protein dysfunction, especially under conditions of glutathione depletion [51]. In addition to its inherent toxicological profile, quercetin may potentiate the toxicity of other substances by either enhancing their bioavailability or through direct synergistic mechanisms of cellular damage [52,53]. In summary, the safety profile of quercetin in humans requires a comprehensive assessment, particularly regarding the threshold for high-dose administration.

## 4. Gut Microbiota and Cancer

### 4.1. Characteristics of Gut Microbiota Dysbiosis in Cancer Patients

Notable disparities exist in the composition of gut microbiota and derived metabolites between cancer patients and healthy adults (Table 2). Firstly, the gut microbiota of cancer patients demonstrates reduced α and β diversity, which is negatively correlated with both the earlier age of onset and the advanced stage of cancer [59,60,61]. In addition, despite common variations among cancer patients, studies have observed the enrichment of some “cancer alliance” microorganisms. A notable illustration of this phenomenon is *Fusobacterium nucleatum*, which drives cancer progression through two key synergistic mechanisms: direct cancer cell modulation (via adhesins such as FadA that activate oncogenic β-catenin and NF-κB signaling) and shaping an immunosuppressive tumor microenvironment (by recruiting MDSCs and inhibiting T-cell-mediated cytotoxicity) [62,63,64,65,66,67,68]. Other carcinogenic species contain *Enterococcus faecalis* [69,70], *Bacteroides fragilis* [71,72], *Escherichia coli* [73,74], *Helicobacter pylori* [75], *Peptostreptococcus anaerobius* [76,77], etc. Surprisingly, these harmful microorganisms have the ability to colonize the tumor microenvironment by disrupting the mucosal barrier, migrating along physiological duct structures, and relying on blood dissemination [78]. Hence, the detection and elimination of carcinogenic bacteria is a promising direction for cancer prevention and treatment. At the metabolic level, the gut microbiota dysbiosis displays an upregulated sulfur metabolism profile, leading to an increase in genotoxic hydrogen sulfide (H_2_S) production [79]. Besides, enhanced conjugated bile acid anabolism is observed in some cancer patients, which is positively correlated with a high-fat diet and obesity [80,81]. Enrichment of polyamine metabolism, which supports cancer progression, has also been detected in the gut microbiota of cancer patients [82,83]. In contrast, short-chain fatty acids (SCFAs), which exhibit a range of health benefits and are generated through the fermentation of dietary fiber by gut microbiota, are markedly reduced in cancer patients [60,84,85]. As a result, it is intriguing to ponder whether alterations in the gut microbiota of cancer patients impair quercetin metabolism. Despite the lack of direct evidence, it has been reported that the species responsible for quercetin metabolism, such as *Lactococcus* and *Akkermansia muciniphila*, are mostly downregulated in cancer patients [86,87,88]. From a logical perspective, reduced utilization of quercetin by the gut microbiota can attenuate the inhibitory effect of dietary quercetin on tumor progression.

### 4.2. Targeted Modulations of Gut Microbiota

**Figure 2:**
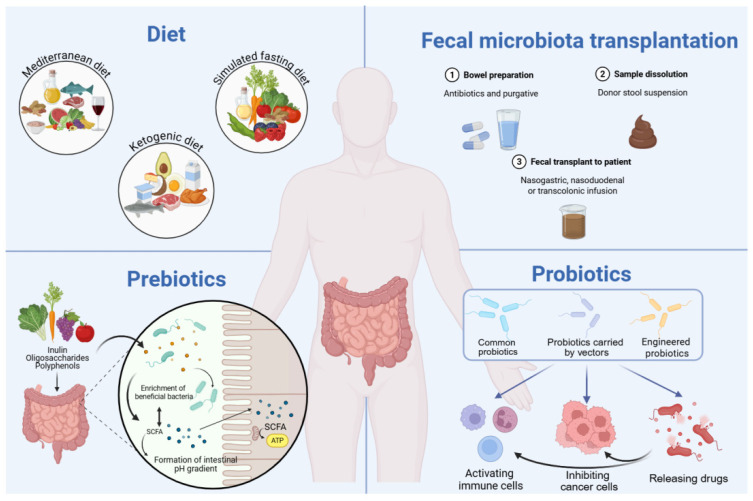
Methods for modulating gut microbiota: fecal microbial transplantation, probiotics and prebiotics, and dietary intervention.

Based on evidence of gut microbial dysbiosis, it is reasonable to consider interventions aimed at restoring the balance of the gut microbiota as a potential adjunct to cancer treatment therapy [9,89]. Given that the pathways through which disturbed gut microbiota influence cancer have been extensively discussed in previous reviews, this section focuses on the regulatory mechanisms governing gut microbiota. Currently, the primary methods for modulating the gut microbiota include fecal microbiota transplantation (FMT), probiotics and prebiotics, and dietary interventions (Figure 2). Lessons learned from these interventions provide a conceptual framework for optimizing quercetin delivery and for stratifying patients according to microbiome features that may predict response.

#### 4.2.1. FMT

FMT refers to the transplantation of the gut microbiota from a healthy donor to a diseased patient to achieve whole gut microbiota replacement and cure diseases [90]. Compared with traditional antibiotic applications, FMT can effectively enrich the diversity of intestinal microbes and exhibit positive, favorable outcomes in clinical investigations [91]. In particular, FMT has been included in clinical guidelines for the treatment of recurrent *Clostridioides difficile* infection [92,93]. In 2021, two studies were conducted to test for the first time whether FMT could improve the response to anti-PD-1 immunotherapy in refractory melanoma patients. The results indicated that part of refractory melanoma patients achieved clinical remission after receiving responder-derived FMT in conjunction with immunotherapy, yielding response rates of 30% (3 of 10) and 40% (6 of 15), respectively [93,94]. Heightened abundance of specific taxa associated with anti-PD-1 response and beneficial immune cell changes were observed in responding patients, consistent with the findings of animal model experiments [95,96]. In 2023, another study evaluated the effective response of untreated patients with advanced melanoma who were administered health donor-derived FMT and anti-PD-1 immunotherapy [97]. The objective response rate was 65% (13 of 20), which was superior to the reported clinical outcome of anti-PD-1 immunotherapy alone. Furthermore, FMT is also beneficial for the management of immune-related adverse events (irAEs). Halsey et al. found that FMT increased the α-diversity of gut microbes and the abundances of *Collinsella* and *Bifidobacterium* in refractory immune checkpoint inhibitor-induced colitis patients, ultimately achieving a clinical remission of 92% [98]. However, the extensive implementation of FMT continues to encounter several challenges. The first issue is the absence of a unified and standardized process setting for FMT [90]. Besides, the assurance of safety and efficacy is compromised due to the limitations of detection technologies and the intricate composition of the gut microbiota [99]. Of note, it has recently been reported that the regional microbiome mismatches resulting from FMT may lead to detrimental metabolic and immune consequences in the recipients. To address the above concerns, the next phase of FMT as cancer treatment therapy should prioritize improving the influencing factors of implementation procedures and exploring the feasibility of combining with other microbial interventions [100].

#### 4.2.2. Probiotics and Prebiotics

A probiotic is a functional concept defined as live microorganisms that confer health benefits to the host when consumed in sufficient amounts. Probiotics primarily belong to the *Lactobacillus* and *Bifidobacterium* genera and are obtained from either healthy individuals or fermented foods [101]. Numerous experiments document the impact of probiotic supplementation on the composition of gut microbiota and the levels of microbiome-derived metabolites, as well as anti-cancer effects. For example, *Lacticaseibacillus rhamnosus* feeding significantly increased the alpha and beta diversity of the gut microbiota and shifted towards beneficial microbial dominance. Interestingly, the modulatory effects of probiotics on gut microbiota are intrinsically linked to enhanced quercetin metabolism. Specific probiotic strains, such as *Lactobacillus* and *Bifidobacterium* species, express functional enzymes such as β-glucosidases, which directly hydrolyze quercetin glycosides into bioactive aglycones [88,102]. Moreover, probiotics drive a broader microbial shift toward quercetin-metabolizing taxa (such as *Akkermansia muciniphila*), further amplifying the biotransformation capacity [87]. At the metabolic level, the accumulation of SCFA and alterations in other metabolites (including α-KG, N-acetyl-l-glutamic acid, and pyridoxine) were detected, thereby promoting the activation of CTLs and the inhibition of Tregs in the tumor microenvironment [103]. Another study demonstrated that oral administration of *Lactobacillus acidophilus* inhibited the growth of hepatocellular carcinoma by increasing pentanoate production and subsequent inhibition of the GPR41/43-Rho-GTPase pathway [104]. In recent years, in order to overcome the shortcomings of low colonization, researchers have developed more advanced application tools based on probiotics platforms. Embedding probiotics with delivery systems represents the most prevalent option. Current delivery systems include nanomaterials [105,106], liposomes [107,108], probiotic spores, and so on [109]. Strikingly, the delivery systems have been upgraded to target metabolic features (ROS and metabolic waste accumulation) within the tumor microenvironment, enabling a “release switch” configuration [105,110]. In addition, some research groups focus on employing synthetic biology and biomedical engineering techniques to modify probiotics, resulting in the development of “engineered probiotics.” There are two main functions of engineered probiotics: (1) directly interfering with the metabolic process of cancer cells and restoring tumor immune responses [111,112,113], and (2) acting as carriers that target the tumor microenvironment to deliver anti-cancer drugs and synergistically eliminate cancer cells [114,115,116]. Despite the limited number of advanced probiotics that have progressed to clinical trials, this nascent field demonstrates great potential for innovation [117,118].

Prebiotics broadly refer to non-digestible dietary components, which can selectively stimulate the growth and activity of beneficial gut microbes, thereby providing a range of health advantages [119]. With the emphasis on the regulatory function of the gut microbiota, the scope of prebiotics has expanded to include more than traditional oligosaccharide carbohydrates, such as plant polyphenols [120]. In the colon, prebiotics are utilized and fermented by specific microorganisms, serving not only to supply energy but also to generate advantageous metabolite SCFAs [121]. Interestingly, SCFAs and other acidic byproducts are involved in intestinal pH gradient formation, and variations in pH in turn influence the fermentation process. As in vitro simulation experiments have shown [122], a pH of 6.5 favored *Bacteroides* to produce propionate, while a pH reduction to 5.5 resulted in a greater tendency to produce butyrate. Undoubtedly, the enhancement of beneficial bacteria and SCFAs is the foundational element and critical connection of the prebiotics anti-cancer function. Han et al. developed an inulin gel that could stay in the colon for a longer period of time, and detected changes in microbiota using 16S ribosomal RNA gene sequencing. The results showed an increase in *Akkermansia* and a decrease in lipopolysaccharide-producing *Oscillibacter*, accompanied by an increase in SCFAs. The anti-tumor mechanism of inulin synergistically enhancing anti-PD-1 treatment was further explored, which relies on the activation of CD8 T cells via SCFAs and receptor GPR43 [123]. In addition to the regulation of immunity, SCFAs also exert direct inhibitory effects on cancer cells. Typically, after being converted to acetyl-CoA by β-oxidation, SCFAs enter the TCA cycle to provide energy for normal mammalian cells [124]. However, cancer cells preferentially consume glucose, a phenomenon known as the Warburg effect [125]. This metabolic preference results in the accumulation of SCFAs and inhibits the proliferation of cancer cells by acting as inhibitors of histone deacetylases (HDACs) [126]. Moreover, by activating the sensing receptors expressed on cancer cells, SCFAs suppress multiple oncogenic signaling pathways and cancer development [127,128,129]. However, the role of prebiotics is closely related to the state of the gut microbiota, which has higher requirements for the application. Personalizing the type and dosage of prebiotics according to the patient’s intestinal microbial characteristics is a problem that remains to be solved.

#### 4.2.3. Diet

A reasonable and balanced diet is the fundamental basis for the preservation of human health. Based on a large number of experimental and clinical studies, several specific dietary patterns have been identified [130]. While dietary patterns exhibit differing capacities to influence the gut microbiota, they collectively have positive implications for cancer [131,132]. The ketogenic diet is made up of low carbohydrates, moderate amounts of protein, and high fat, forcing the body to consume fat and produce ketones [133,134]. A reduction in the Firmicutes phylum/Bacteroidetes phylum ratio was observed in participants on a ketogenic diet using 16S rRNA gene sequencing, with the most significant decline occurring in Bifidobacterium [135]. Another investigation examined the long-term impacts of the ketogenic diet on the composition of gut microbiota. In week 12, there was a statistically significant difference in the β-diversity of gut microbes. Similarly, the ketogenic diet decreased the relative abundance of Bifidobacteria at the genus level, beginning in week 4 and persisting through week 12 [136]. Besides, the ketogenic diet induced a shift in gut microbiota towards stearate accumulation, involving an increase in bacterial species such as *Phocea* spp. and *Akkermansia* spp. and depletion of *Barnesiella* spp. and others. Stearate facilitated apoptosis of cancer cells and a decrease in Th17 immune cells [137]. The Mediterranean diet represents the eating style of southern European countries along the Mediterranean coast, which is dominated by vegetables, fruits, whole grains, and olive oil. Consequently, the Mediterranean diet is rich in active ingredients such as polyunsaturated fatty acids ω-3, Phenolic compounds, and fiber, which have the effect of increasing both the α-diversity and metabolic activity of the gut microbiota [138,139]. Even in regions that are distant from the gastrointestinal tract, the microbiota inside them is regulated by the Mediterranean diet. Shively et al. reported that, compared to Western dietary feeding, Mediterranean diet feeding promoted Lactobacillus dominant growth in the mammary gland and observed an increase in bile acid metabolites and bacterial-processed bioactive compounds [140]. Symbiotic microbiota changes induced by the Mediterranean diet prevent the development of cancer mainly by attenuating systemic inflammatory responses (reducing the permeability of the intestinal barrier to LPS) and increasing antioxidative stress capacity [138,141]. It is noteworthy that quercetin, along with other polyphenols, constitutes a significant component of the Mediterranean diet, primarily derived from sources such as vegetables, fruits, and legumes [142]. In contrast, the ketogenic diet prioritizes high-fat intake while strictly limiting carbohydrate consumption, resulting in notably lower quercetin intake. This difference may, in part, explain the disparities between these two dietary patterns in terms of their regulatory effects on the gut microbiota and their respective anticancer efficacy [143]. In addition, the fasting-mimicking diet has recently made tremendous progress in cancer treatment, mainly achieved by limiting caloric intake to mimic the effects of fasting [144]. Ke et al. revealed that the fasting-mimicking diet enriched *Bifidobacterium pseudolongum*, which increased the attack of tissue-resident memory CD8 T-cell population on cancer cells by producing L-arginine [145]. The modulation of the gut microbiota by the fasting-mimicking diet varies depending on design schemes. Another fasting regimen (three days fasting, four days of feeding, three weeks of repetition) raised butyrate-producing bacteria and butyrate concentrations in mouse models. Elevated butyrate upregulated pan-Kcr expression and suppressed pancreatic cancer cell proliferation [146]. Fasting-mimicking diet treatment (four days of FMD diet followed by three days of unrestricted normal diet) also provided a protective effect for *Lactobacillus* [147]. Dietary interventions in gut microbiota have the advantages of simplicity, economy, and ease of operation, but whether cancer patients can tolerate potential adverse effects and achieve long-term management needs validation through more extensive data [148,149]. Besides, these dietary patterns are typically rich in plant polyphenols, including quercetin, which may partly mediate microbiota-dependent cancer-protective effects. However, the specific contribution of quercetin within these complex dietary exposures remains largely undefined.

## 5. The Influence of the Interaction Between Quercetin and Gut Microbiota on Cancer

Although the focus of current research is the direct anti-tumor mechanism of quercetin, with the exploration of the interaction between quercetin and the gut microbiota, the role of the gut microbiota in the antitumor effects of quercetin has garnered significant scholarly interest. On the one hand, the gut microbiota expresses a diverse array of enzymes capable of metabolizing quercetin, which not only takes part in the biotransformation of quercetin but also exerts inhibitory effects on tumor cells by producing quercetin-derived metabolites.

### 5.1. Metabolism of Quercetin by the Gut Microbiota

Similar to the metabolic processes in the stomach and small intestine, quercetin glycosides and quercetin conjugates that reach the colonic lumen are initially hydrolyzed by extracellular glycosidases secreted by microorganisms, resulting in the release of quercetin aglycones [15,150]. Rutin (Q-3-*O*-rutinoside), in particular, predominantly relies on the gut microbiota for degradation, as the human body does not possess the rhamnose-glucose deglycosylation enzymes [151]. Mediated by bacterial α-l-rhamnosidase and β-glucosidases, rutin is approximately transformed into isoquercetin (quercetin-3-*O*-glucoside) or quercetin aglycone [152,153]. However, only a limited number of bacterial species genomes express α-l-rhamnosidase, and there exist variations in both the metabolic rates and the metabolite composition. One study isolated and identified 10 bacterial strains capable of metabolizing rutin from healthy human fecal samples, mainly belonging to *Enterococcus*, *Lactococcus*, and *Escherichia* genera. Bacterial strains from the genus *Lactococcus* produced quercetin with the highest efficiency, while genus *Enterococcus* were more inclined to yield isoquercetin, which was positively correlated with l-rhamnosidase activity [88]. Surprisingly, none of the 33 human strains of genus *Bifidobacterium* demonstrated the ability to hydrolyze rutin, although the genomes of *Bifidobacterium catenulatum* and *Bifidobacterium pseudocatenultum* contained α-l-rhamnosidase sequence and could remove rutinose from hesperidin. This indicates that the expression level of α-l-rhamnosidase is not a definitive predictor of rutin hydrolytic ability [102]. Furthermore, an alternative research approach involves the analysis and comparison of the microbiota composition among donors exhibiting differing responses to rutin. The results showed that individuals exhibiting high levels of quercetin production were characterized by an increased abundance of *Lachnospiraceae* (*Lachnoclostridium* and *Eisenbergiella*) [154]. *Enterobacteriaceae* were enriched in high isoquercetin producers. There were also studies examining rutin fermentation by the microbiota residing in different intestinal segments within the same individual. The colonic microbiota has an increased fermentation rate of rutin relative to the ileum, attributable to the higher prevalence of genes encoding α-rhamnosesidase and β-glucosidase. Ileal fermentation accounted for about 20% of rutin, and the proportion was related to the abundance of Firmicutes [155]. In addition to the microbiota, the deconjugation of quercetin glycosides is also influenced by the sugar moiety, which encompasses the number of sugar moieties and the pattern and position of the hydroxyl group, as well as the type of glycosidic bond (*C*- or *O*-glycoside) [156]. Then, catalyzed by microbial enzymes, quercetin aglycones experience ring fission, accompanied by catabolic processes such as hydroxylation, methylation, decarboxylation, and the shortening of side chains, and are ultimately metabolized into a series of phenolic acids [157,158,159]. For example, Wang et al. found 11 derivatives of benzoic acid, phenylacetic acid, and phenyl propionic acid in the cecum contents after the introduction of quercetin aglycone and quercetin-3-*O*-sophoroside into the cecum [160]. 3,4-dihydroxyphenylacetic acid, 3-methoxy-4-hydroxyphenylacetic acid, and 3-hydroxyphenylacetic acid were detected in the urine of healthy volunteers after ingestion of tomato juice containing rutin, with concentrations corresponding to 22% of the rutin intake [161]. Analogous to the first step, a small fraction of gut microbiota is responsible for the generation of phenolic acids. Ranjini et al. screened five bacterial species that could degrade quercetin from 94 human gut bacteria, namely *Bacillus glycinifermentans*, *Flavonifractor plautii*, *Bacteroides eggerthii*, *Olsenella scatoligenes,* and *Eubacterium eligens* [162]. *Clostridium perfringens* and *Bacteroides fragilis* have also been demonstrated to convert quercetin to 3,4-dihydroxyphenylacetic acid, with the highest conversion efficiency at the concentration of 1 mg/mL quercetin in broth [163]. Interestingly, the fermentation activity of *Bacteroides uniformis* on quercetin-3-*O*-rutinose-7-*O*-α-l-rhamnoside promoted the proliferation of *Akkermansia muciniphila*. In *B. uniformis* culture, quercetin was transformed into taxifolin, whereas the quercetin metabolites identified in the coculture of *B. uniformis* and *A. muciniphila* included taxifolin and kaempferol. Thus, quercetin metabolism is not only the result of the activity of a particular single bacterial species [87]. Besides, the appropriate concentrations of bile acids and glucose confer a competitive advantage and improve metabolic capacity for the microbiota involved in the metabolism of quercetin [164,165]. To accurately explore the actual fermentation process, it is essential to develop novel experimental systems that can effectively mimic the in vivo conditions present within the intestinal lumen.

### 5.2. Anti-Cancer Mechanisms of Microbial Phenolic Acids

The observation of diminished microbial phenolic acid production in the colon of cancer patients and the protective effects of phenolic acid-rich diet against cancer suggest that these compounds may possess anti-cancer properties [166,167]. In fact, phenolic acids exert a variety of anti-cancer functions: arresting cell cycle/proliferation, inducing apoptosis, inhibiting EMT, reducing COX-2 activity and expression, enhancing cancer immunity, and reversing drug resistance (Table 3) [162,168].

The cell cycle is comprised of four distinct phases: the G1 phase (pre-DNA synthesis), S phase (DNA synthesis phase), G2 phase (late DNA synthesis phase), and M phase (mitotic phase), which are precisely controlled by the interplay of cyclins, cyclin-dependent kinases (CDKs), and cyclin-dependent kinase inhibitors (CKIs) [186]. Due to aberrant expression of regulatory factors, cancer cells exhibit cell cycle dysregulation and unlimited proliferation [187,188]. Thus, the block of the cell cycle can effectively target proliferating active cancer cells. Phenolic acids produced by microbial metabolism, consistent with quercetin, negatively inhibit the cell cycle of cancer cells. In colon adenocarcinoma Caco-2 and SW480 cell lines, 3-(3′-hydroxyphenyl) propanoic acid, 3-(3′-hydroxyphenyl) acetic acid, and 3-(3′,4′-dihydroxyphenyl) acetic acid treatments induced an increase in the percentage of S phase and a decrease of G_2_/M phase, indicating cell cycle arrest in the S phase [17]. Similarly, 3,4-dihydroxyphenylacetic acid exerts an inhibitory effect on cell cycle progression in the S phase by upregulating genes which are known to promote cell cycle arrest and apoptosis, such as *EGR1* and *TNF*, in HT-29 cell lines [169]. Further, 3,4-dihydroxyphenylacetic acid also demonstrated the ability to reduce cell viability and manipulate cell cycle arrest in the G_0_/G_1_ phase in HT-29 cell lines [170]. Moreover, despite the absence of observed accumulation in a specific phase of the cell cycle, 2,4,6-trihydroxybenzoic acid impaired the proliferation and colony formation of SLC5A8-pLVX cells and MDA-MB-231 cells. This effect relied on direct suppression of CDK 1, 2, and 4 enzyme activity and promotion of CDK inhibitory proteins p21^Cip1^ and p27^Kip1^ expression by 2,4,6-trihydroxybenzoic acid after cellular uptake via SLC5A8 [171]. Besides, the growth of cancer tissues depends not only on unlimited proliferation, but also on resistance to the apoptosis program. Specific mechanisms have been identified, including altered equilibrium between pro-apoptotic and anti-apoptotic proteins, diminished caspase activity, and compromised signaling through death receptors [189,190]. Among various phenolic acids, 3,4-dihydroxybenzoic acid displays the most pronounced pro-apoptotic effect. Research indicated that 3,4-dihydroxybenzoic acid led to apoptosis in OVCAR-3 cells, through the activation of PARP and caspase-3, the upregulation of Bax, and the downregulation of Bcl-2 [172]. Further, 3,4-dihydroxybenzoic acid also curbed HO-1 expression, which catalyzed heme catabolism and generated antioxidant products, thereby inducing oxidative stress and apoptosis in Caco-2 cells [173]. JNK/p38 MAPK signaling activation and Fas-mediated caspase activation were also potential targets for 3,4-dihydroxybenzoic acid, inducing apoptosis in AGS cells [174]. During cancer progression, some cancer cells acquire the ability to invade locally and infiltrate the circulatory system for metastasis [1]. Aberrant reactivation of epithelial-mesenchymal transition (EMT) is a fundamental process that involves the remodeling of adhesion molecules and the cytoskeleton. Ujlaki et al. [191] identified the regulatory effect of bacterial metabolites on EMT in 4T1 murine breast cancer cells and found that 3-hydroxyphenylacetic acid and 4-hydroxybenzoic acid decreased the expression of EMT markers (such as vimentin and Snail) and the proportion of the mesenchymal cells. However, the underlying mechanism and the reason for the V-shaped dose-response curve have not been revealed [175]. Surprisingly, 3,4-dihydroxybenzoic acid could inhibit EMT in A549 and H1299 cells even under induction conditions of TGF-β by abrogating the activation of PI3K/Akt/mTOR signaling pathway [176]. In animal experiments, 3,4-dihydroxybenzoic acid also targeted RhoB and hindered the Ras/Akt/NF-κB pathway, thereby blocking metastasis of B16/F10 melanoma cells to the liver [177]. Cyclooxygenase-2 (COX-2) facilitates the transformation of arachidonic acid into prostaglandins primarily in the context of inflammatory processes. Notably, COX-2 and its downstream metabolite Prostaglandin E_2_ were upregulated in cancer tissues, which supported immune evasion and cancer progression [192,193]. Experiments indicated that phenolic acids are natural inhibitors of COX-2. Ana and her colleagues evaluated the binding affinity of 3-Hydroxyphenylacetic and 4-hydroxyphenylpropionic acid toward COX-2 through docking analysis and found that dianionic ligands directly inhibited the activity of COX-2 [178]. Furthermore, although the production of PGE_2_ remained unchanged, five phenolic acids, such as 3,4-dihydroxyphenylacetic acid and 3-(3,4-dihydroxyphenyl)-propionic acid, reduced the expression of COX-2 and hindered the induction of COX-2 by TNF-α [179,194]. In comparison to NSAIDs, phenolic acids have a higher safety profile and a reduced incidence of adverse effects. The immune system can recognize and eliminate mutant cells, a process called immune surveillance. However, after undergoing immune editing, cancer cells successfully attain the objective of immune escape [195]. Based on this fact, restoring the attack of immune cells against cancer cells is at the core of cancer immunotherapy. Phenolic acids contribute to the enhancement of anti-tumor immunity through the modulation of immune cell function and cytokine levels. Furthermore, 3,4-dihydroxyphenylacetic acid and 4-hydroxyphenylacetic acid stimulation improved the cytotoxic activity of macrophages against A375 cells and counteracted heme-induced suppression. Activated macrophages after stimulation upregulated the activity of hexokinase and pyruvate kinase, glycolysis, HIF-α levels, and release of IP-10 [180]. Also, 500 ppm 3,4-dihydroxybenzoic acid administration significantly raised IFN-γ and SMAD4 levels in primary cultured human natural killer cells, which was beneficial for shaping the anti-inflammatory tumor microenvironment [181]. In terms of cytokines, benzoic acid, phenylacetic acid, and phenylpropionic acid lessened the expression of IL-8, TNF-α, and VCAM-1 in Caco-2 cells and had a synergistic effect with SCFAs [182]. In female Balb/c mice after receiving probiotic Canan SV-53 and 3,4-dihydroxybenzoic acid treatment, there was a notable elevation in serum IgA levels and the population of IgA-producing B cells within the ileum. Concurrently, a reduction in the pro-inflammatory cytokines IL-17A, IL-6, and IL-23 was documented [183]. Finally, drug resistance is the main factor in the failure of cancer chemotherapy. P-glycoprotein expression is an important target of multidrug resistance and functions as an ATP-dependent transporter that can excrete chemotherapy drugs from cells, thereby diminishing intracellular drug levels. Experiments demonstrated that living multidrug-resistant K562/Dox cell lines treated with 4-hydroxybenzoic acid and 4-hydroxy-3-methoxybenzoic acid regained sensitivity to pirarubicin, which was associated with impaired P-glycoprotein function. The specific mechanism was ATP deficiency, as 4-hydroxybenzoic acid and 4-hydroxy-3-methoxybenzoic acid decreased ΔΨm, SDH activity, and mitochondrial function [184]. Moreover, 4-hydroxybenzoic acid also reversed adriamycin resistance in human breast cancer cells by inhibiting HDAC6 activity and promoting p53 and HIPK2 expression [185].

**Figure 3:**
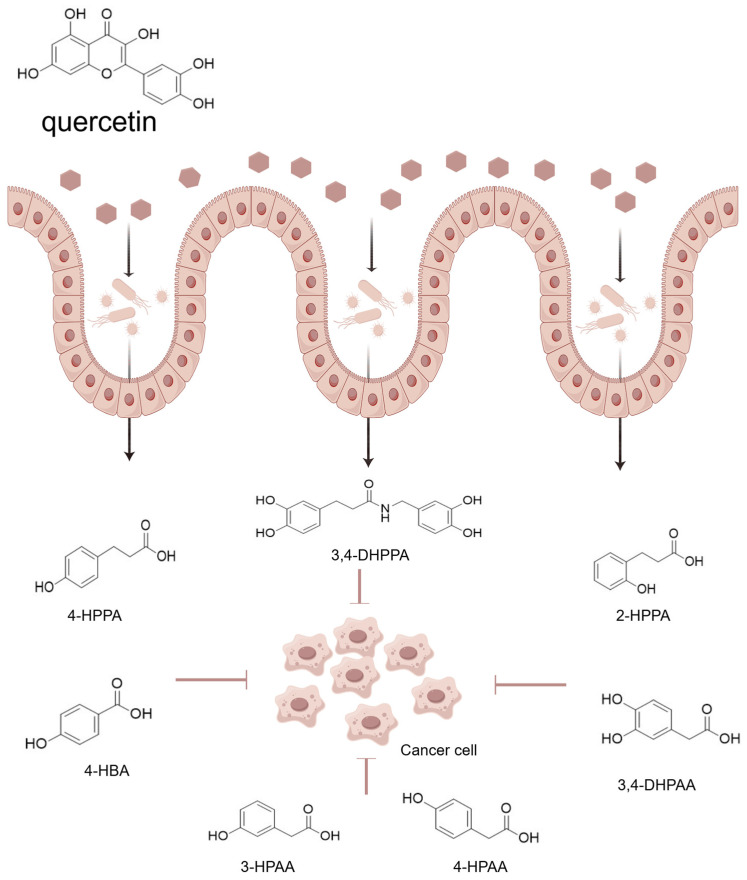
Quercetin metabolism by gut microbiota in the colon and the anti-cancer effects of phenolic acid metabolites.

Overall, adequate evidence to date supports the potential of microbial phenolic acids as a therapeutic agent in cancer treatment. Supplementation of phenolic acids through diet and the metabolism of gut microbiota may be more effective than the administration of individual phenolic acids, as the antitumor mechanisms of phenolic acids of different structures are different and may have synergistic interactions (Figure 3).

### 5.3. Anti-Cancer Modification of the Gut Microbiota by Quercetin

**Figure 4:**
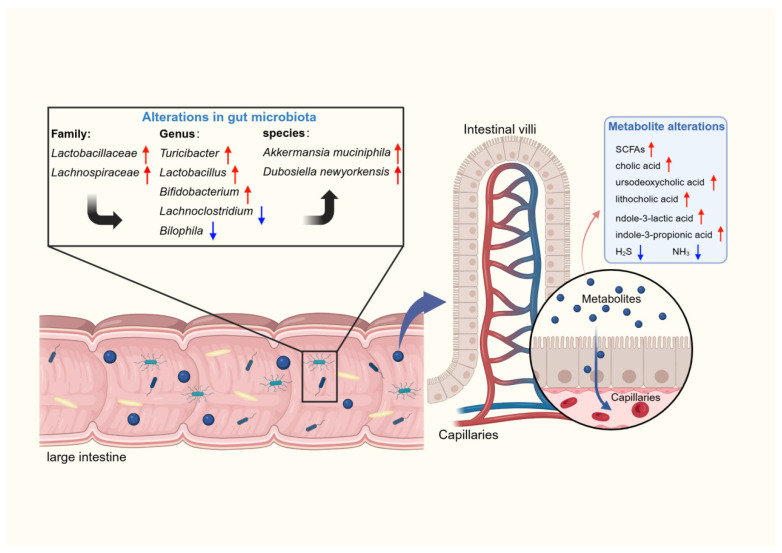
Other beneficial bacterial candidates for quercetin-responsive anti-cancer targets. Chematic illustration of the modulatory effects of quercetin on gut microbiota composition across three levels (family, genus, species) and their associated metabolic profiles. Red arrows indicate upregulation, and blue arrows indicate downregulation.

During the past several years, the modification of gut microbiota by quercetin has been increasingly recognized (Figure 4). Functionally, quercetin can be categorized as a prebiotic, as evidenced by its effects on increasing the diversity of the microbial community, elevating the relative abundance of beneficial bacteria, and altering the microbial metabolic profile [196,197,198]. Moreover, modifications of the gut microbiota are indispensable mechanisms for the positive effects of quercetin on cancer. Comprehending microbial modifications paves the way for expanding anti-cancer applications of quercetin.

Several recent studies have explored quercetin-induced structural alterations in gut microbiota and potential anti-cancer mechanisms. Manni et al. found that quercetin administration significantly increased the β-diversity of the gut microbiota and induced the enrichment of beneficial bacterial species, especially *Akkermansia muciniphila*. Microbial transformation potentiated the inhibitory efficacy of cyclophosphamide in triple-negative breast cancer models, which was mediated through a synergistic increase in the systemic populations of CD4^+^ T cells and NK cells, concomitant with a reduction in Treg cells [18]. Wu et al. examined changes in the gut microbiota and HCC following quercetin treatment. 16S rRNA sequencing revealed a restoration of gut microbiota imbalance, manifested by elevated abundances of *Firmicutes*, *Actinomycetes*, and *Verrucomicrobia* at the phylum level, alongside an increase of *Dubosiella* and *Akkermansia* at the species level. The combined application of quercetin and anti-PD-1 antibody enhanced the cytotoxic activity of macrophages and reshaped the cancer microenvironment, resulting in a marked suppression of HCC progression [199]. Gao et al. extracted natural exosome-like lipid nanoparticles rich in quercetin glycosides (rutin and quercetin 3-*O*-glucoside) from the black mulberry leaves, which had superior anti-HCC outcomes through direct cancer cell killing and gut microbiota regulation. Increased Chao and Shannon diversity index, reduced *Firmicutes*/*Bacteroidetes* ratio, and relative enrichment of *Lactobacillus* and *Turicibacter* were observed within the gut microbiota, all of which have been linked to the suppression of cancer progression [200]. Although quercetin interventions yielded divergent microbial outcomes, this variability can be tentatively explained by dosage, treatment duration, and the chemical structure of quercetin glycosides [16]. Furthermore, beyond the above-mentioned genera and species, there are other beneficial bacteria as candidates for anti-cancer targets in response to quercetin. Guo et al. revealed that green pea hull extract feeding, mainly composed of quercetin and other common polyphenols, promoted the growth of *Lactobacillaceae* and *Lachnospiraceae* and ultimately alleviated DSS-induced colitis in mice models [201]. In previous studies, the *Lachnospiraceae* family was found to be significantly reduced in tumor tissue. Supplementation of *Ruminococcus gnavus* and *Blautia producta* in the *Lachnospiraceae* family could degrade lyso-glycerophospholipids and restore the anti-cancer function of CD8^+^ T cells [202]. Pan et al. reported the modulation of gut microbiota by in vitro fermentation of nine flavonoids, including rutin, isoquercetin, and quercetin [198]. At the genus level, the relative abundance of *Bifidobacterium* and *Lactobacillus* was notably elevated, and their mechanisms of anti-cancer activity have been thoroughly elucidated. Conversely, the potentially pathogenic *Lachnoclostridium* and *Bilophila* were reduced, which were novel bacterial markers for cancer [95,203,204,205,206].

In addition to changes in the structure of gut microbiota, quercetin also induces the remodeling of microbial metabolic profiles. SCFAs, deficient in the gut microbiota of cancer patients, have been shown to be response molecules to quercetin. Mi et al. found that 0.2% quercetin-containing AIN-93G improved the disruption of the gut microbiota by antibiotics, mainly by the enrichment of butyrate-producing bacteria and the enhancement of butyrate production pathways (mainly lysine pathways) [207]. Ghimire et al. demonstrated that quercetin significantly increased *Acidaminococcus intestini* and iso-butyrate production. The combination of quercetin and prebiotic rice bran mainly increased propionate levels, which was associated with a decrease in the potentially pathogenic *Enterobacteriaceae* family [208]. Bile acids play a dual role in cancer progression. The biosynthesis of bile acids involves two pathways: the classical pathway to produce cholic acid (CA), and the alternative pathway to produce chenodeoxycholic acid (CDCA), which then enter the intestine and undergo microbial metabolism to form secondary bile acids such as deoxycholic acid (DCA), lithocholic acid (LCA), and ursodeoxycholic acid (UDCA) [209]. Experiments revealed that quercetin treatment accelerated the conversion of cholesterol to CA in a way that relied on increased *A. Muciniphila* abundance and sterol 12α-hydroxylase expression [210]. Quercetin treatment also facilitated the production of non-12α-hydroxylated BAs (UDCA and LCA) by enriching *Lactobacillus* [211]. Quercetin-driven remodeling of bile acid metabolism not only inhibits cancer development but also improves obesity and metabolic status to support cancer prevention [212,213,214]. In addition, through the feeding of quercetin, the gut microbiota enhanced tryptophan metabolism and increased levels of the metabolites indole-3-lactic acid and indole-3-propionic acid [210,215]. Tryptophan metabolites have potent anti-cancer immune cell stimulatory effects through epigenetic mechanisms and the activation of Aryl hydrocarbon receptor, suggesting that these metabolites likely contribute to the anti-cancer effects of quercetin [216,217,218].

## 6. Enhancement Strategies for Quercetin-Gut Microbiota Interaction in Cancer and Future Challenges

Due to the anti-cancer effect of phenolic acids, enhancing the metabolic efficiency of quercetin by gut microbiota is of great significance. First of all, the structure and physicochemical properties of quercetin glycosides are important influencing factors. As described in the metabolic process, the sugar moiety of quercetin glycosides greatly influences metabolism rate and bioavailability [156]. In addition, water-soluble αG-rutin demonstrated faster absorption and maintained higher plasma concentrations relative to rutin and quercetin, indicating that structural modification aimed at increasing solubility represents an optional strategy [219]. Secondly, for colorectal diseases, especially CRC prevention and treatment, advances in quercetin delivery systems can provide abundant quercetin glycoside substrates for gut microbiota, improving the instability and loss of quercetin glycosides. At present, the majority of carrier materials are selected from natural polysaccharides and prebiotics due to their favorable biocompatibility and reliance on microbial degradation. Zhang et al. employed porous starch to adsorb quercetin and embedded it in the CMC matrix to form hydrogel beads, which were pH-sensitive and effectively released quercetin under simulated intestinal conditions at a pH of 7.4. Jing et al. [220]. achieved the release of quercetin in a manner responsive to both pH and the colon microenvironment by formulating microspheres using pectin and Ca^2+^ and crosslinking with oligochitosan [221]. In the presence of natural polysaccharides and prebiotics, the microbial community responsible for quercetin metabolism also exhibited enhanced efficiency in producing phenolic acids. Quercetin loaded with alginate microspheres, supplemented with inulin and chitosan as a protective coating, produced higher levels of 3-HPPA, 3-HPAA, 3,4-DHPA, and 4-HPA relative to the administration of quercetin in its unencapsulated form. In the microsphere group, microbial diversity was enhanced, with a notable increase in the relative abundance of *Lactobacillus*, *Turicibacter*, *Eubacterium*, and *Clostridium*, alongside a reduction in *Enterococcus* levels, which collectively promoted phenolic acid production [222]. Besides, nanotechnology has also been applied to the targeted delivery of quercetin to the colon, but there is no evidence that nano-delivery promotes microbial metabolism of quercetin [223,224]. Finally, modulating the transformation of gut microbiota composition to quercetin hypermetabolism can significantly decrease drug dosage and toxic side effects. Different bacterial species possess distinct enzymatic capabilities for the degradation of quercetin, and this microbial variability explains the inconsistencies in quercetin metabolism observed between individuals. Fortunately, interventions targeting the gut microbiota are well established and have made progress. In vitro experiments demonstrated that the addition of common prebiotics, such as inulin, markedly enhanced the fermentation of rutin by fecal microbiota, especially 3,4-dihydroxyphenylacetic acid levels, which increased by 2.5-fold [225]. However, concurrent administration of prebiotics may impede the interaction between quercetin and the gut microbiota, potentially causing a delay or inhibition in the generation of phenolic acids [226]. Collectively, the literature presents a complex and sometimes contradictory picture regarding the efficacy of prebiotics in enhancing quercetin metabolism. These apparent discrepancies are not uncommon in microbiome research and likely stem from several critical variables. The type and structural specificity of the prebiotic, its administered dose, and the duration of intervention can differentially shape microbial community structure and function. More fundamentally, the baseline composition and functional capacity of an individual’s gut microbiota—a major source of inter-individual variation—determine whether a given prebiotic will enrich taxa capable of metabolizing quercetin. Additionally, experimental conditions and the potential for substrate competition between the prebiotic and quercetin glycosides for microbial utilization may further influence outcomes. Therefore, a blanket statement on the effect of prebiotics is not warranted. Instead, the current evidence supports a context-dependent view: prebiotics can enhance quercetin metabolism, but their success is contingent upon a favorable alignment of prebiotic properties, host microbial ecology, and intervention parameters. Additionally, probiotics are also a frequently employed combined strategy to boost phenolic acid production. Quercetin and probiotics exhibit a synergistic relationship: some probiotic strains express quercetin metabolism-related enzymes to support quercetin biotransformation, and quercetin creates environmental advantages conducive to probiotic growth and colonization [227,228]. In summary, strategies to improve microbial metabolism of quercetin are continually advancing. Most studies now quantify this metabolic process primarily by measuring the levels of phenolic acids in plasma or feces. However, the absence of assessment of the gut microbiota and metabolic enzymes may lead to reduced accuracy and reproducibility. Furthermore, integrating phenolic acid production patterns with microbiota analysis may guide patient stratification and outcome prediction.

The modulation of gut microbiota by quercetin is also affected by a variety of factors. It is reasonable that increasing the level and duration of quercetin in the colon can amplify microbial alteration. Hu et al. prepared quercetin and HP-β-CD inclusion complex gels, which have the advantages of intestinal release and sustained-release effect. Correspondingly, inclusion complex gels more effectively restore the diversity of gut microbiota and lower the *Firmicutes*/*Bacteroidetes* value, thereby mitigating brain injury induced by cancer radiation therapy [229]. Besides, quercetin and probiotics or other prebiotics may have a synergistic effect in microbial alteration, since these interventions are both able to drive a shift in the gut microbiota towards anti-cancer phenotypes. The combination of microencapsulated probiotics (*Bifidobacterium bifidum* and *Lactobacillus gasseri*) and quercetin significantly reduced the number of aberrant crypt foci and adenomas by inhibiting the Wnt/β-catenin signaling pathway in Apc^Min/+^ mice compared to application alone. At the microbial level, only the combination treatment group observed an increase in *Bifidobacterium* and *Lactobacillus* population and a decrease in *Enterobacteriaceae*, verifying the synergistic effect on the gut microbiota [230]. In addition, Qi et al. demonstrated that alternating feeding of β-glucan and quercetin increased *Parabacteroides* abundance, decreased TNF-α levels, and downregulated the expression of inflammatory and cancer-related genes (*Hmgcs2*, *Fabp2*, and *G*pt) in mice with CRC [231]. The above studies reveal that improving quercetin-induced modification of gut microbiota is effective in boosting cancer suppression outcomes. More application details and mechanisms need to be confirmed due to differences in the gut microbiota of human and animal models and potential side effects.

## 7. Conclusions and Future Perspectives

Quercetin has explicit anti-cancer function and exhibits an easy-access and safe profile. However, quercetin treatment is also constrained by its inherent instability and low bioavailability [232]. Besides, the involvement of the gut microbiota in cancer represents an emerging research frontier. The dysbiosis of gut microbiota observed in cancer patients is mainly characterized by reduced α/β-diversity, relative enrichment of cancer-associated microorganisms, and decreased beneficial SCFAs. Despite the development of strategies aimed at regulating the gut microbiota, adverse events have been documented in clinical applications, and achieving sustained long-term management remains challenging [90,233]. Fortunately, recent findings reveal the molecular crosstalk between quercetin and the gut microbiota, offering a novel improvement strategy. The gut microbiota is responsible for metabolizing quercetin into phenolic acids, enhancing quercetin utilization and effective anti-cancer concentrations. Quercetin treatment induced a shift in the gut microbiota of cancer patients from dysbiosis to an anti-cancer phenotype, marked by enhanced microbial diversity, increased abundance of beneficial species, and elevated levels of SCFAs. To enhance the interaction between quercetin and the gut microbiota, the main approach is to modify the structure of quercetin, use carriers to deliver quercetin, or combine quercetin with other interventions for the gut microbiota. Specifically, the synergistic application of quercetin and probiotics, as functional foods or nutraceutical formulations, has been widely developed and evaluated for efficacy [234,235,236]. The synergistic anti-cancer mechanisms of quercetin and probiotics include that probiotics are beneficial for the growth of quercetin-metabolizing species, quercetin reshapes the gut microbiota to create a favorable colonization environment for probiotics, jointly promoting the anti-cancer phenotype of the gut microbiota and synergistically inhibiting cancer cell proliferation or enhancing anti-cancer immune responses.

In the future, more details regarding the crosstalk between quercetin and the gut microbiota are worth further exploration. First of all, specific gut microbial species involved in the metabolism of quercetin remain unidentified. Moreover, the metabolic activity of quercetin in certain species is also influenced by other species and environmental factors. Detecting the metabolic level of the gut microbiota prior to quercetin administration can be used to identify responsive patients and predict prognosis. Besides, quercetin is frequently co-administered with probiotics or prebiotics, which may theoretically interfere with the contact of quercetin with the gut microbiota. In order to achieve the maximum therapeutic effect, optimizing the timing of administration and reasonably choosing the types of probiotics or prebiotics are critical challenges in this domain. In addition, the production of phenolic acids and the alterations in the gut microbiota are diverse and exhibit individual differences, resulting in fluctuations in the final anti-cancer effects. Although the role of the gut microbiota in the anti-cancer function of quercetin cannot be ignored, there are currently only a few clues. Therefore, although the role of the gut microbiome in the anti-cancer function of quercetin cannot be ignored, current clues are limited and preliminary. To date, while the gut microbiota’s role in mediating quercetin’s anti-cancer effects is not to be overlooked, the evidence supporting this interaction remains limited and largely preliminary.

## Figures and Tables

**Table 1:** The anti-tumor effects of quercetin.

Dose	Administration	Tumor	Animals	Mechanism	Ref.
2.5 mg/kg	i.v. (IRQL/CPP liposomes)	glioblastoma tumor (U87 xenografts)	Female BALB/c nude mice	(−) HSP70, (−) HIF-1α, (+) apoptosis, (−) proliferation	Das S et al., 2025 [54]
10, 20 mg/kg	i.v. (Q-MX-ZMOF@CH)	Breast tumor (MCF-7 xenografts)	Female BALB/c nude mice	(−) HSP70, (−) HSP90, (−) HSP27	Elbeltagi S et al., 2025 [55]
50 mg/kg	i.p.	Stomach tumor (MKN28 xenografts)	BALB/c nude mice	(−) proliferation, (+) apoptosis, (+) autophagy, (+) HIF-1α accumulation, (−) Akt–mTOR signaling, (+) BNIP3/BNIP3L	Wang K et al., 2011 [56]
10, 20 mg/kg	i.v. (R-D-Q micelles)	Breast tumor (MCF-7 xenografts)	Female BALB/c nude mice	(+) p-p38, (+) p-JNK, (−) p-ERK, (+) apoptosis	Yang C et al., 2025 [57]
5 mg/kg	i.p.	Prostate cancer (22RV1 xenografts)	Male BALB/c nude mice (immunocompromised Nu/Nu)	(−) IQGAP1, (−) ANGPTL4–IQGAP1–Raf–MEK–ERK–PGC1α signaling, (−) OXPHOS	Xiong Z et al., 2025 [44]
75 mg/kg	i.p.	Prostate cancer (PC-3 and LNCaP xenografts)	Male BALB/c nude mice	(+) apoptosis, (−) proliferation, (−) PI3K/Akt signaling, (−) AR/PSA signaling, (−) EMT and stem-like phenotype	Lu X et al., 2020 [42]
--	i.v. (AHA@MnP/QCT)	Lung tumor (LLC xenografts)	C57BL/6 mice	(+) apoptosis, (+) ferroptosis, (+) ICD	Qiu C et al., 2025 [58]

**Table 2:** The characteristics of gut microbiota dysbiosis in cancer patients.

Cancer Type	Gut Microbiota or Metabolite	Changing Trends	Ref.
CRC	α-diversity, short-chain fatty acid and GABA biosynthesis	↓	Kong C et al., 2023 [60]
CRC	Fusobacterium nucleatum, tryptophan, bile acid and choline metabolism	↑	Kong C et al., 2023 [60]
CRC	Fusobacterium nucleatum	↑	Yu T et al., 2017 [62]
CRC	pks Escherichia coli	↑	Jans M et al., 2024 [73]
CRC	Peptostreptococcus anaerobius	↑	Liu Y et al., 2024 [76]
CRC	H_2_S production	↑	Wolf PG et al., 2022 [79]
CRC	polyamines (cadaverine and putrescine)	↑	Yang Y et al., 2019 [82]
CRC	SCFAs	↓	Alvandi E et al., 2022 [84]
CRC	SCFAs	↓	Yang Y et al., 2023 [85]
BC	Bacteroides fragilis	↑	Iida N et al., 2021 [69]
BC	Fusobacterium nucleatum	↑	Jans M et al., 2024 [73]
HCC	Enterococcus faecalis	↑	Parhi L et al., 2020 [68]
ESCC	Fusobacterium nucleatum	↑	Zhang JW et al., 2023 [65]
PDAC	α-diversity and β-diversity	↓	Hong J et al., 2024 [59]

Note: “↑” for upregulation (increased expression); “↓” for downregulation (decreased expression); CRC, Colorectal Cancer; BC, Breast Cancer; HCC, Hepatocellular Carcinoma; ESCC, Esophageal Squamous Cell Carcinoma; PDAC, Pancreatic Ductal Adenocarcinoma.

**Table 3:** Anti-cancer mechanisms of microbial phenolic acids.

Models	Type of Phenolic Acids	Mechanisms and Effects	Ref.
In Caco-2 and SW480 cell lines	3,4-DHPA, 3-HPAA, and 3-HPPA	arrest the cell cycle at the S phase and anti-proliferative	Cattivelli A et al., 2023 [17]
In HCT-116 cell lines	2,4,6-THBA, 3,4-DHBA, 3,4,5-THBA, and 3,4-DHPA	anti-proliferative and inhibit colony formation ex vivo	Sankaranarayanan R et al., 2021 [162]
In HT-29 cell lines	3,4-DHPA	arrest the cell cycle at the S phase by upregulating cell cycle arrest and apoptosis genes	García-Gutiérrez N et al., 2023 [169]
In HT-29 cell lines	3,4-DHPA	reduce cell viability and manipulate cell cycle arrest in the G0/G1 phase	Rosa LS et al., 2018 [170]
In SLC5A8-pLVX cells and MDA-MB-231 cells	2,4,6-THBA	directly suppress of CDK 1, 2 and 4 enzyme activity and promote CDK inhibitory proteins p21Cip1 and p27Kip1 expression	Henning SM et al., 2013 [171]
In OVCAR-3 cells	PCA	facilitate apoptosis through the activation of PARP and caspase-3, the upregulation of Bax, and the downregulation of Bcl-2	Xie Z et al., 2018 [172]
In Caco-2 cells.	PCA	curb HO-1 expression and induce oxidative stress and apoptosis	Acquaviva R et al., 2021 [173]
In AGS cells	PCA	activate the JNK/p38 MAPK signaling and Fas caspase-mediated apoptosis	Lin HH et al., 2007 [174]
In 4T1 murine breast cancer cells	3-HPAA and 4-HBA	decrease the expression of EMT markers (such as vimentin and Snail) and the proportion of the mesenchymal cells	Ujlaki G et al., 2023 [175]
In A549 and H1299 cells	PCA	inhibit EMT by abrogating the activation of PI3K/Akt/mTOR signaling pathway	Yang MH et al., 2021 [176]
In B16/F10 melanoma cells	PCA	target RhoB and hinder Ras/Akt/NF-κB pathway to block metastasis	Lin HH et al., 2011 [177]
--	3-HPAA and 4-HPPA	directly bind and inhibit the activity of COX-2	Amić A et al., 2016 [178]
In HT-29 cell lines	3,4-DHPA, 3-HPAA, and 3,4-diHPP	reduce the expression of COX-2 and hinder the induction of COX-2 by TNF-α	Karlsson PC et al., 2005 [179]
In RKO cells	3-HPAA	hinder malignant transformation and mitochondrial dysfunction induced by hemin	Catalán M et al., 2020 [168]
In A375 cells	3,4-DHPA and 4-HPA	improve the cytotoxic activity of macrophages and counteract heme-induced suppression	Carrasco-Pozo C et al., 2020 [180]
In ApcMin/+ Mice	3,4-DHBA	raise IFN-γ and SMAD4 levels in NK cells and shape anti-inflammatory tumor microenvironment	Dong A et al., 2022 [181]
In TNF-α-induced Caco-2 cell model	BzA, PAA, and PPA	lessen the expression of IL-8, TNF-α and VCAM-1 and have a synergistic effect with SCFAs	Zheng S et al., 2021 [182]
In female Balb/c mice	PCA	elevate serum IgA levels and the population of IgA-producing B cells within the ileum, and reduce the pro-inflammatory cytokines IL-17A, IL-6, and IL-23	Shahbazi R et al., 2023 [183]
In multidrug-resistant K562/Dox cell lines	4-HBA and VA	decrease ΔΨm, SDH activity, and mitochondrial function, thereby inhibiting ATP-dependent P protein function	Myint O et al., 2022 [184]
In human breast cancer cells	4-HBA	reverse adriamycin resistance by inhibiting HDAC6 activity and promoting p53 and HIPK2 expression	Wang XN et al., 2018 [185]

## Data Availability

No new data were created or analyzed in this study. Data sharing is not applicable to this article.

## Abbreviations

BI, Bioaccessibility Index; SCFAs, short-chain fatty acids; FMT, fecal microbiota transplantation; CRC, colorectal cancer; BC, breast cancer; HCC, hepatocellular carcinoma; ESCC, esophageal squamous cell carcinoma; PDAC, pancreatic ductal adenocarcinoma; EMT, epithelial-mesenchymal transition; COX-2, cyclooxygenase-2; HSP70, heat shock protein 70; HIF-1α, hypoxia-inducible factor 1-alpha; HDACs, histone deacetylases; CDKs, cyclin-dependent kinases; CKIs, cyclin-dependent kinase inhibitors; PARP, poly (ADP-ribose) polymerase; MAPK, mitogen-activated protein kinase; JNK, c-Jun N-terminal kinase; ERK, extracellular signal-regulated kinase; PI3K, phosphoinositide 3-kinase; Akt, protein kinase B; mTOR, mechanistic target of rapamycin; NF-κB, nuclear factor kappa-light-chain-enhancer of activated B cells; TNF-α, tumor necrosis factor-alpha; IL, interleukin; IFN-γ, interferon-gamma; TGF-β, transforming growth factor-beta; VEGF, vascular endothelial growth factor; LPS, lipopolysaccharide; H2S, hydrogen sulfide; GABA, γ-aminobutyric acid; PGE2, prostaglandin E2; PD-1, programmed cell death protein 1; CTLs, cytotoxic T lymphocytes; Tregs, regulatory T cells; NK cells, natural killer cells; irAEs, immune-related adverse events; ICD, immunogenic cell death; DSS, dextran sulfate sodium; 3-HPAA, 3-hydroxyphenylacetic acid; 4-HPAA, 4-hydroxyphenylacetic acid; 3,4-DHPAA, 3,4-dihydroxyphenylacetic acid; 4-HBA, 4-hydroxybenzoic acid; 3,4-DHPPA, 3-(3,4-dihydroxyphenyl)propionic acid; PCA, protocatechuic acid; VA, vanillic acid; HVA, homovanillic acid.
